# Impact of the COVID-19 pandemic on the emergency transportation of older patients: a population-based descriptive study in Osaka prefecture, Japan

**DOI:** 10.3389/fpubh.2025.1515635

**Published:** 2025-10-03

**Authors:** Kenta Tanaka, Yusuke Katayama, Tetsuhisa Kitamura, Hisaya Domi, Jun Oda, Tetsuya Matsuoka

**Affiliations:** ^1^The Working Group to Analyze the Emergency Medical Care System in Osaka Prefecture, Osaka, Japan; ^2^Division of Environmental Medicine and Population Sciences, Department of Social and Environmental Medicine, Osaka University Graduate School of Medicine, Suita, Japan; ^3^Department of Traumatology and Acute Critical Medicine, Osaka University Graduate School of Medicine, Suita, Japan; ^4^Osaka Prefectural Government, Osaka, Japan; ^5^Rinku General Medical Center, Izumisano, Japan

**Keywords:** COVID-19 pandemic, death, emergency transportation, incidence, older patients

## Abstract

**Background:**

This study aimed to assess the influence of the COVID-19 pandemic on emergency medical services for older patients who were transported to hospitals by ambulance.

**Methods:**

This descriptive retrospective study was conducted between January 2019 and December 2022, using the Osaka Emergency Information Research Intelligent Operation Network system. All patients aged ≥65 years who were transported by ambulance to hospitals in Osaka Prefecture for acute diseases were included. The outcomes were the number of older patients transported by ambulance, the number of difficulties obtaining patient acceptance at hospitals, and the number of deaths following hospitalization. We calculated the incidence rate ratio (IRR) and its associated 95% confidence interval (CI) for each year of the study period (2020, 2021, and 2022) using Poisson regression, with 2019 as the control year.

**Results:**

Compared to 2019, the numbers of older patients transported for acute disease were 186,218 (IRR, 0.92; 95% CI: 0.92–0.93) in 2020, 186,955 (0.93, 0.92–0.93) in 2021, and 214,048 (1.06, 1.05–1.07) in 2022. Difficulty obtaining patient acceptance increased over time (6,668 in 2020, 9,894 in 2021, and 22,790 in 2022). The number of deaths were 8,660 (1.01, 0.98–1.04) in 2020, 9,754 (1.14, 1.10–1.17) in 2021, and 11,050 (1.29, 1.25–1.32) in 2022.

**Conclusion:**

The number of older patients transported to emergency departments in Osaka Prefecture for acute diseases increased in 2022 compared with 2019. Difficulties obtaining patient acceptance and in-hospital deaths also increased over this period.

## 1 Introduction

Coronavirus disease 2019 (COVID-19) caused by Severe Acute Respiratory Syndrome Coronavirus 2 (SARS-CoV-2) was first identified in China in December 2019, before the infection spread rapidly worldwide (1). On March 11, 2020, the World Health Organization officially declared COVID-19 a pandemic (2). Although there were several known risk factors for developing severe COVID-19, older age was considered the primary one (3, 4). Mortality from COVID-19 was higher in older patients, and mortality increased with age (5, 6).

A number of reports have indicated that the COVID-19 pandemic had significant impacts on emergency medical services (EMSs) worldwide, often comprising longer transport times and worse outcomes following hospitalization (7–10). In Singapore during the pandemic (January–May 2020), the patients waited longer for EMS and pre-hospital return of spontaneous circulation was less likely (9). In Korea, multivariable logistic regression analysis showed that the COVID-19 pandemic was significantly associated with higher in-hospital mortality among patients in the emergency department (10). Older adults tend to make up a large proportion of patients in emergency departments. In Osaka Prefecture, Japan, for example, those aged ≥65 years account for approximately 60% of all patients transported to hospital emergency departments (11). Therefore, it may be important to evaluate the overall EMS system with a focus on older patients, as this group are generally more vulnerable than other age groups to health problems, yet may not have been transported quickly and appropriately to hospitals during the COVID-19 pandemic.

Osaka Prefecture is the largest metropolitan area in western Japan, with a population of approximately 8.8 million people. The annual number of ambulance calls in this region was 500,000 in 2019 (12, 13). The Osaka Emergency Information Research Intelligent Operation Network (ORION) system, a population-based emergency transport registration system in Osaka, includes pre-hospital care records and post-transport outcomes (14). This study aimed to describe the actual situation regarding emergency transportation of older patients to hospitals by EMS before and after the COVID-19 pandemic in Osaka Prefecture, and to assess the effect of the pandemic on this type of transportation, using the ORION database.

## 2 Materials and methods

### 2.1 Study design, population, and setting

This was a descriptive retrospective study with a period spanning January 1, 2019, to December 31, 2022. We used the database of the population-based registry of emergency department patients, comprising both ambulance and in-hospital records managed by ORION. This database is operated by Osaka Prefecture and covers all patients transported to critical care centers and emergency hospitals in the prefecture. Information concerning the ORION database has been previously described in detail (14, 15). Briefly, the ambulance crew at the scene operate the ORION smartphone application for each emergency patient. All data entered into the mobile app are recorded, including vital signs and the time between receipt of the request and admission to the hospital. The smartphone app data are then stored on the ORION cloud server. Cooperating with the dispatched ambulance crew, the data manager at each fire department directly enters and uploads the ambulance records for each emergency patient, which can then be linked to the app data. Operators at each hospital also directly input or upload patient data, such as diagnoses and outcomes, after patients are admitted, and the results compiled by the ORION system are fed back to each fire department and emergency hospital. The ORION system is installed in all fire departments and emergency hospitals in Osaka Prefecture. Its data are administrative records that are anonymized without specific personal information such as patient names, birth dates, and addresses. Therefore, the requirement to obtain informed patient consent was waived for this study. This study was approved by the ethics committee of Osaka University Graduate School of Medicine (approval no.: 15003). All procedures were conducted in accordance with the Declaration of Helsinki.

In 2020, 8,837,685 people lived in the 1905 km^2^ area of Osaka Prefecture. Of these, 2,441,984 (27.6%) were aged ≥65 years (12). This study defined older patients as those aged 65 years or older, according to the protocol used in previous related studies, and focused on this population (16).

### 2.2 Measurements

Reasons for ambulance calls were divided into: “fire accident,” “natural disaster,” “water accident,” “traffic accident involving car, ship, or aircraft,” “injury, poisoning, and disease caused by industrial accident,” “disease and injury caused by sports,” “other injury,” “trauma caused by assault,” “self-induced injury,” “acute disease,” and “others” (14, 15). Patients who were transported as inter-hospital relocations were excluded. We focused on patients whose reasons for calling an ambulance were “acute disease” and who were successfully transported and admitted to a hospital. We also focused on any difficulties patients experienced obtaining acceptance to hospitals, and defined such cases as those in which ambulance crews made ≥4 phone calls to the hospitals before obtaining patient acceptance, as well as those in which the crews stayed at the scene for >30 min, based on national standards (17). Post-hospitalization diagnoses were classified according to International Classification of Diseases, 10th Revision codes (ICD-10), which classified COVID-19 as category “U” (18). For the “surge” periods during which the COVID-19 epidemic spread through the region, Osaka Prefecture recorded the first surge between January 29, 2020 and June 13, 2020; second surge between June 14, 2020 and October 9, 2020; third surge between October 10, 2020 and February 28, 2021; fourth surge between March 1, 2021 and June 20, 2021; fifth surge between June 21, 2021 and December 16, 2021; sixth surge between December 17, 2021 and June 24, 2022; seventh surge between June 25, 2022 and September 26, 2022; and eighth surge between September 27, 2022 and May 8, 2023 (19).

### 2.3 Outcomes

The outcome measures of this study were the number of older patients transported to emergency departments for acute disease, the number of deaths among them, the number of difficulties obtaining patient acceptance from hospitals, and the time interval from ambulance call to arrival on scene. The patients’ outcomes after 21 days of hospitalization were also recorded and classified into four categories: continued hospitalization, discharged, changed hospital, and death.

### 2.4 Statistical analysis

Based on previous related studies, we used the 2019 outcome measures as a reference (i.e., the control period before the COVID-19 pandemic) and compared them by year and month for 2020 (the first year of the pandemic), 2021 (the second year), and 2022 (the third year) (11). We calculated the incidence rate ratio (IRR) and its associated 95% confidence interval (CI) by year and month for 2020, 2021, and 2022 using a Poisson regression model with 2019 serving as the control year and the census population of Osaka Prefecture in 2020 as the denominator (12). This is because the number of emergency transports in Osaka Prefecture was considered to be very low relative to its population. As there were no cases with an ICD-10 diagnosis of “U” in 2019, the IRRs for 2021 and 2022 were calculated based on 2020 for these cases. Difference in the time intervals from ambulance call to arrival on scene by year was assessed using a Jonckheere-Terpstra test. All statistical analyses were performed using STATA MP version 16.0 (StataCorp, College Station, TX, USA).

## 3 Results

**Figure 1:**
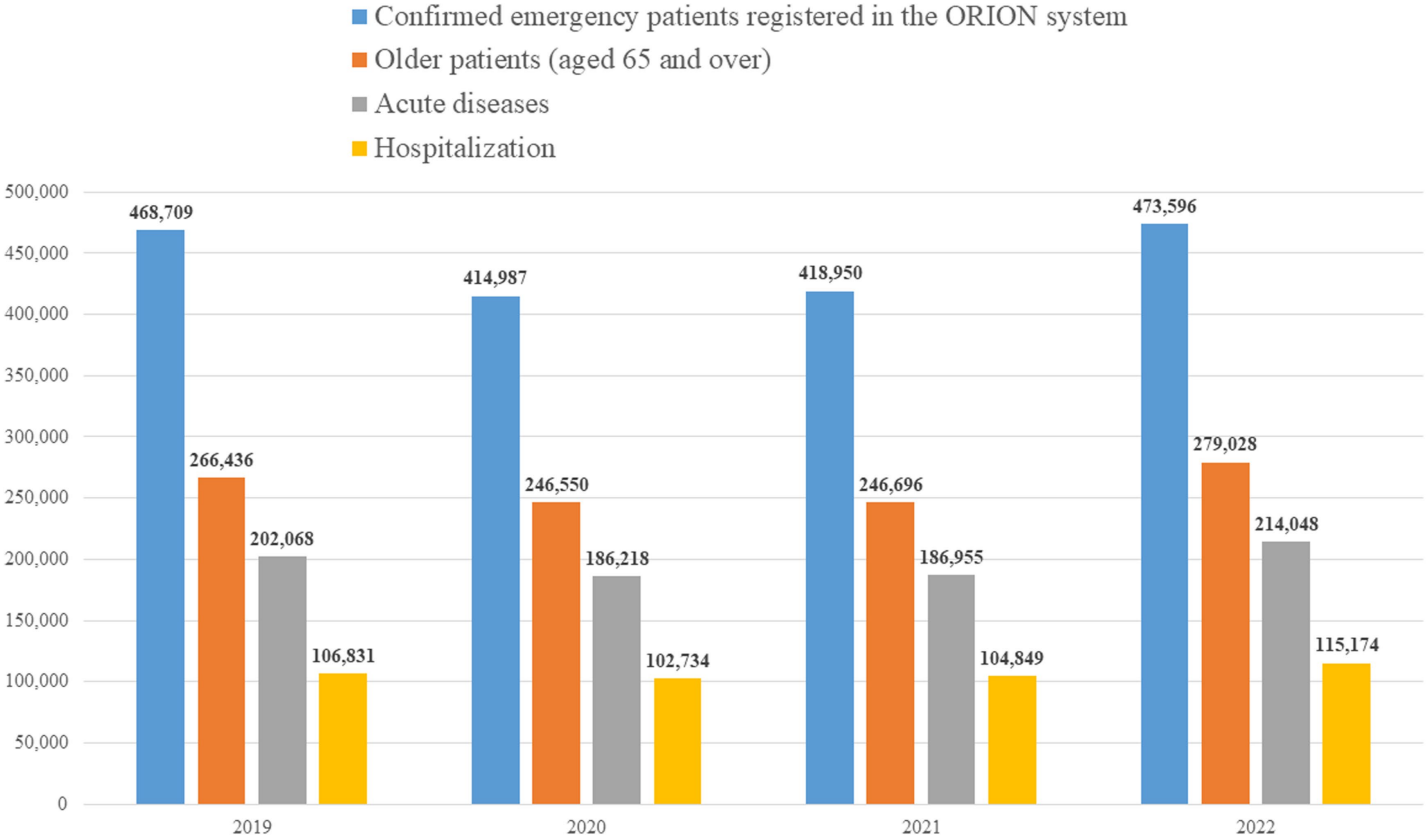
Flow chart.

The numbers of older patients were 266,436 in 2019, 246,550 in 2020, 246,696 in 2021, and 279,028 in 2022 (Figure 1). The mean and standard deviation of the time intervals from ambulance call to arrival on scene were 7.4 ± 2.7 min in 2019, 7.5 ± 2.7 min in 2020, 7.9 ± 3.0 min in 2021, and 8.8 ± 4.4 min in 2022 and it increased significantly over year (*P* for trend < 0.05).

Table 1 shows the number of older emergency patients and IRRs (with associated 95% CIs) in each year, by reason for the ambulance call, in 2019–2022. For “acute disease” as the reason for calling an ambulance, the number of emergency patients decreased significantly in 2020 (IRR: 0.92, 95% CI: 0.92–0.93) and in 2021 (IRR: 0.93, 95% CI: 0.92–0.93) compared with 2019; however, it increased significantly in 2022 (IRR: 1.06, 95% CI: 1.05–1.07).

**Table 1:** The number of emergency patients registered in the Osaka emergency information research intelligent operation network system in 2019–2022.

Reason for ambulance call	2019	2020	IRR^a^ (95% CI)	2021	IRR^b^ (95% CI)	2022	IRR^c^ (95% CI)
Fire accident	153	146	0.95 (0.76–1.21)	140	0.92 (0.72–1.16)	134	0.88 (0.69–1.11)
Natural disaster	8	10	1.25 (0.44–3.64)	18	2.25 (0.93–5.98)	2	0.25 (0.03–1.25)
Water accident	21	18	0.86 (0.43–1.69)	25	1.19 (0.64–2.24)	15	0.71 (0.34–1.45)
Traffic accident involving car, ship, or aircraft	10,145	9,046	0.89 (0.87–0.92)*	8,778	0.87 (0.84–0.89)*	9,371	0.92 (0.90–0.95)*
Injury, poisoning, and disease caused by industrial accident	833	690	0.83 (0.75–0.92)*	692	0.83 (0.75–0.92)*	695	0.83 (0.75–0.92)*
Disease and injury caused by sports	129	72	0.56 (0.41–0.75)*	81	0.63 (0.47–0.84)*	109	0.84 (0.65–1.10)
Other injury	52,143	49,460	0.95 (0.94–0.96)*	49,173	0.94 (0.93–0.95)*	53,827	1.03 (1.02–1.04)*
Trauma caused by assault	426	396	0.93 (0.81–1.07)	361	0.85 (0.73–0.98)*	353	0.83 (0.72–0.96)*
Self-induced injury	435	444	1.02 (0.89–1.17)	438	1.01 (0.88–1.15)	426	0.98 (0.85–1.12)
Acute disease	2,02,068	1,86,218	0.92 (0.92–0.93)*	1,86,955	0.93 (0.92–0.93)*	2,14,048	1.06 (1.05–1.07)*
Other	75	50	0.67 (0.46–0.97)*	35	0.47 (0.30–0.71)*	48	0.64 (0.44–0.93)*
Total	2,66,436	2,46,550	0.93 (0.92–0.93)*	2,46,696	0.93 (0.92–0.93)*	2,79,028	1.05 (1.04–1.05)*

IRR, incident rate ratio; CI, confidence interval. ^a^2020 versus 2019. ^b^2021 versus 2019.^c^2022 versus 2019.*P < 0.05.

The number of older emergency patients transported for acute disease, their admissions, and the IRRs (95% CIs) for each month in 2019–2022 are shown in Table 2. Significant increases in older emergency patients transported for acute diseases were seen in July 2022, which corresponds to the seventh COVID-19 surge in Japan, when compared to July 2019, and December 2022, which corresponds to the eighth surge, when compared to December 2019. The number of hospitalized older patients transported for acute disease decreased significantly in 2020 (IRR: 0.96, 95% CI: 0.95–0.97) and 2021 (IRR: 0.98, 95% CI: 0.97–0.99), compared to 2019; however, it increased significantly in 2022 (IRR: 1.08, 95% CI: 1.07–1.09). In 2022, significant increases were observed in almost every month compared with that in 2019.

**Table 2:** The number of patients transported to emergency departments for acute diseases and hospitalized in Osaka in 2019–2022.

Status		January	February	March	April	May	June	July	August	September	October	November	December	Total
Acute disease of transported patients	2019	20,870	15,840	15,957	15,653	16,214	15,415	16,765	18,926	16,385	15,943	16,507	17,593	2,02,068
	2020	18,785	16,023	15,091	13,177	13,500	14,145	15,040	18,419	15,356	15,369	15,064	16,249	1,86,218
	IRR^a^ (95% CI)	0.90 (0.88–0.92)*	1.01 (0.99–1.03)	0.95 (0.92–0.97)*	0.84 (0.82–0.86)*	0.83 (0.81–0.85)*	0.92 (0.90–0.94)*	0.90 (0.88–0.92)*	0.97 (0.95–0.99)*	0.94 (0.92–0.96)*	0.96 (0.94–0.99)*	0.91 (0.89–0.93)*	0.92 (0.90–0.94)*	0.92 (0.92–0.93)*
	2021	17,015	14,222	15,748	14,843	14,155	14,355	16,533	15,929	14,684	16,064	15,827	17,580	1,86,955
	IRR^b^ (95% CI)	0.82 (0.80–0.83)*	0.90 (0.88–0.92)*	0.99 (0.97–1.01)	0.95 (0.93–0.97)*	0.87 (0.85–0.89)*	0.93 (0.91–0.95)*	0.99 (0.97–1.01)	0.84 (0.82–0.86)*	0.90 (0.88–0.92)*	1.01 (0.99–1.03)	0.96 (0.94–0.98)*	1.00 (0.98–1.02)	0.93 (0.92–0.93)*
	2022	19,533	16,599	16,531	15,984	16,413	16,861	20,049	19,758	16,909	17,149	17,268	20,994	2,14,048
	IRR^c^ (95% CI)	0.94 (0.92–0.95)*	1.05 (1.03–1.07)*	1.04 (1.01–1.06)*	1.02 (1.00–1.04)*	1.01 (0.99–1.03)	1.09 (1.07–1.12)*	1.20 (1.17–1.22)*	1.04 (1.02–1.07)*	1.03 (1.01–1.05)*	1.08 (1.05–1.10)*	1.05 (1.02–1.07)*	1.19 (1.17–1.22)*	1.06 (1.05–1.07)*
Hospitalization for acute disease	2019	10,448	8,266	8,606	8,487	8,749	8,292	8,942	9,620	8,676	8,597	8,841	9,307	1,06,831
	2020	9,760	8,582	8,334	7,409	7,538	8,135	8,454	9,640	8,379	8,757	8,528	9,218	1,02,734
	IRR^a^ (95% CI)	0.93 (0.91–0.96)*	1.04 (1.01–1.07)*	0.97 (0.94–1.00)	0.87 (0.85–0.90)*	0.86 (0.84–0.89)*	0.98 (0.95–1.01)	0.95 (0.92–0.97)*	1.00 (0.97–1.03)	0.97 (0.94–1.00)*	1.02 (0.99–1.05)	0.96 (0.94–0.99)*	0.99 (0.96–1.02)	0.96 (0.95–0.97)*
	2021	9,668	8,099	8,945	8,707	8,251	8,010	8,872	8,792	8,192	8,934	8,796	9,583	1,04,849
	IRR^b^ (95% CI)	0.93 (0.90–0.95)*	0.98 (0.95–1.01)	1.04 (1.01–1.07)*	1.03 (1.00–1.06)*	0.94 (0.92–0.97)*	0.97 (0.94–1.00)	0.99 (0.96–1.02)	0.91 (0.89–0.94)*	0.94 (0.92–0.97)*	1.04 (1.01–1.07)*	0.99 (0.97–1.02)	1.03 (1.00–1.06)*	0.98 (0.97–0.99)*
	2022	10,734	9,010	9,068	8,793	8,929	9,061	10,207	9,967	9,109	9,278	9,693	11,325	1,15,174
	IRR^c^ (95% CI)	1.03 (1.00–1.06)*	1.09 (1.06–1.12)*	1.05 (1.02–1.09)*	1.04 (1.01–1.07)*	1.02 (0.99–1.05)	1.09 (1.06–1.13)*	1.14 (1.11–1.17)*	1.04 (1.01–1.07)*	1.05 (1.02–1.08)*	1.08 (1.05–1.11)*	1.10 (1.07–1.13)*	1.22 (1.18–1.25)*	1.08 (1.07–1.09)*

IRR, incident rate ratio; CI, confidence interval. ^a^2020 versus 2019. ^b^2021 versus 2019. ^c^2022 versus 2019. *P < 0.05.

The number of difficulties obtaining patient acceptance from hospitals increased significantly in 2020 (IRR: 1.43, 95% CI: 1.38–1.49), 2021 (IRR: 2.13, 95% CI: 2.05–2.20), and 2022 (IRR: 4.90, 95% CI: 4.74–5.05) compared to 2019 (Table 3). Compared to 2019, the number of difficulties obtaining patient acceptance from hospitals increased significantly in all months of 2022. This was particularly true in August, corresponding to the seventh surge, and December, during the eighth surge. Similar results were observed for the hospitalized cases.

**Table 3:** Difficulties obtaining patient acceptance for patients transported to emergency departments for acute diseases and subsequently hospitalized in Osaka in 2019–2022.

Status		January	February	March	April	May	June	July	August	September	October	November	December	Total
Acute disease of transported patients	2019	990	510	385	347	331	254	280	368	281	254	315	340	4,655
	2020	557	382	421	734	643	269	392	880	466	396	587	941	6,668
	IRR^a^ (95% CI)	0.56 (0.51–0.62)*	0.75 (0.65–0.86)*	1.09 (0.95–1.26)	2.12 (1.86–2.41)*	1.94 (1.70–2.22)*	1.06 (0.89–1.26)	1.40 (1.20–1.64)*	2.39 (2.11–2.71)*	1.66 (1.43–1.93)*	1.56 (1.33–1.83)*	1.86 (1.62–2.14)*	2.77 (2.44–3.14)*	1.43 (1.38–1.49)*
	2021	1,307	794	649	1,254	1,206	514	623	940	858	586	545	618	9,894
	IRR^b^ (95% CI)	1.32 (1.21–1.44)*	1.56 (1.39–1.74)*	1.69 (1.48–1.92)*	3.61 (3.21–4.08)*	3.64 (3.22–4.13)*	2.02 (1.74–2.36)*	2.23 (1.93–2.57)*	2.55 (2.26–2.89)*	3.05 (2.67–3.51)*	2.31 (1.99–2.68)*	1.73 (1.50–1.99)*	1.82 (1.59–2.08)*	2.13 (2.05–2.20)*
	2022	2,047	3,512	2,401	1,209	983	675	2,226	3,372	1,514	925	1,258	2,668	22,790
	IRR^c^ (95% CI)	2.07 (1.92–2.23)*	6.89 (6.27–7.57)*	6.24 (5.60–6.96)*	3.48 (3.09–3.94)*	2.97 (2.62–3.37)*	2.66 (2.30–3.08)*	7.95 (7.02–9.03)*	9.16 (8.23–10.23)*	5.39 (4.74–6.14)*	3.64 (3.17–4.20)*	3.99 (3.53–4.53)*	7.85 (7.01–8.81)*	4.90 (4.74–5.05)*
Hospitalization for acute disease	2019	551	297	216	189	195	138	156	196	144	128	168	199	2,577
	2020	305	221	248	469	399	158	240	543	284	252	359	616	4,094
	IRR^a^ (95% CI)	0.55 (0.48–0.64)*	0.74 (0.62–0.89)*	1.15 (0.95–1.38)	2.48 (2.09–2.95)*	2.05 (1.72–2.44)*	1.14 (0.91–1.45)	1.54 (1.25–1.89)*	2.77 (2.35–3.28)*	1.97 (1.61–2.43)*	1.97 (1.59–2.45)*	2.14 (1.77–2.58)*	3.10 (2.63–3.65)*	1.59 (1.51–1.67)*
	2021	879	543	408	855	800	323	377	597	562	377	332	402	6,455
	IRR^b^ (95% CI)	1.60 (1.43–1.78)*	1.83 (1.58–2.11)*	1.89 (1.60–2.24)*	4.52 (3.86–5.32)*	4.10 (3.50–4.82)*	2.34 (1.91–2.88)*	2.42 (2.00–2.93)*	3.05 (2.59–3.60)*	3.90 (3.24–4.72)*	2.95 (2.40–3.63)*	1.98 (1.64–2.39)*	2.02 (1.70–2.41)*	2.50 (2.39–2.62)*
	2022	1,260	2,112	1,512	725	606	384	1,246	1,860	941	567	779	1,605	13,597
	IRR^c^ (95% CI)	2.29 (2.07–2.53)*	7.11 (6.29–8.06)*	7.00 (6.07–8.11)*	3.84 (3.26–4.53)*	3.11 (2.64–3.67)*	2.78 (2.28–3.41)*	7.99 (6.76–9.50)*	9.49 (8.19–11.05)*	6.53 (5.48–7.84)*	4.43 (3.65–5.41)*	4.64 (3.92–5.51)*	8.07 (6.96–9.39)*	5.28 (5.06–5.51)*

IRR, incident rate ratio; CI, confidence interval. ^a^2020 versus 2019. ^b^2021 versus 2019. ^c^2022 versus 2019. *P < 0.05.

Table 4 shows the patient outcomes 21 days after hospitalization. The number of discharges decreased significantly in 2020 (IRR: 0.93, 95% CI: 0.92–0.94) and 2021 (IRR: 0.91, 95% CI: 0.90–0.92), compared to 2019; however, it remained comparable in 2022 (IRR: 1.01, 95% CI: 1.00–1.02). The number of continued hospitalizations increased significantly in 2022 compared with 2019 (IRR: 1.11, 95% CI: 1.09–1.12). The number of deaths increased significantly in 2021 (IRR: 1.14, 95% CI: 1.10–1.17) and 2022 (IRR: 1.29, 95% CI: 1.25–1.32). This tendency was seen in almost all months of 2022 compared to the corresponding months in 2019, with particularly higher increases in February, which corresponds to the sixth surge, August, representing the seventh surge, and December, during the eighth surge.

**Table 4:** Outcomes of patients transported to emergency departments in Osaka for acute diseases, at 21 days following hospitalization, in 2019–2022.

Outcomes	January	February	March	April	May	June	July	August	September	October	November	December	Total
Continued hospitalization													
2019	3,167	2,525	2,732	2,712	2,568	2,474	2,625	2,745	2,581	2,606	2,652	2,858	32,245
2020	3,043	2,711	2,623	2,324	2,208	2,433	2,696	2,742	2,562	2,636	2,622	2,858	31,458
IRR^a^ (95% CI)	0.96 (0.91–1.01)	1.07 (1.02–1.13)*	0.96 (0.91–1.01)	0.86 (0.81–0.91)*	0.86 (0.81–0.91)*	0.98 (0.93–1.04)	1.03 (0.97–1.08)	1.00 (0.95–1.05)	0.99 (0.94–1.05)	1.01 (0.96–1.07)	0.99 (0.94–1.04)	1.00 (0.95–1.05)	0.98 (0.96–0.99)*
2021	3,016	2,641	2,824	2,719	2,461	2,421	2,746	2,628	2,565	2,763	2,718	3,031	32,533
IRR^b^ (95% CI)	0.95 (0.91–1.00)	1.05 (0.99–1.11)	1.03 (0.98–1.09)	1.00 (0.95–1.06)	0.96 (0.91–1.01)	0.98 (0.92–1.04)	1.05 (0.99–1.10)	0.96 (0.91–1.01)	0.99 (0.94–1.05)	1.06 (1.00–1.12)*	1.02 (0.97–1.08)	1.06 (1.01–1.12)*	1.01 (0.99–1.02)
2022	3,402	2,858	3,023	2,775	2,716	2,697	3,008	2,908	2,911	2,817	3,111	3,501	35,727
IRR^c^ (95% CI)	1.07 (1.02–1.13)*	1.13 (1.07–1.19)*	1.11 (1.05–1.17)*	1.02 (0.97–1.08)	1.06 (1.00–1.12)*	1.09 (1.03–1.15)*	1.15 (1.09–1.21)*	1.06 (1.01–1.12)*	1.13 (1.07–1.19)*	1.08 (1.02–1.14)*	1.17 (1.11–1.24)*	1.22 (1.17–1.29)*	1.11 (1.09–1.12)*
Discharged													
2019	5,802	4,570	4,676	4,709	4,997	4,768	5,215	5,731	4,963	4,802	4,872	5,136	60,241
2020	5,286	4,564	4,408	3,935	4,126	4,592	4,631	5,600	4,644	4,812	4,474	4,819	55,891
IRR^a^ (95% CI)	0.91 (0.88–0.95)*	1.00 (0.96–1.04)	0.94 (0.90–0.98)*	0.84 (0.80–0.87)*	0.83 (0.79–0.86)*	0.96 (0.92–1.00)	0.89 (0.85–0.92)*	0.98 (0.94–1.01)	0.94 (0.90–0.97)*	1.00 (0.96–1.04)	0.92 (0.88–0.96)*	0.94 (0.90–0.98)*	0.93 (0.92–0.94)*
2021	4,901	4,149	4,624	4,286	4,194	4,374	4,885	4,820	4,350	4,685	4,610	4,937	54,815
IRR^b^ (95% CI)	0.84 (0.81–0.88)*	0.91 (0.87–0.95)*	0.99 (0.95–1.03)	0.91 (0.87–0.95)*	0.84 (0.81–0.87)*	0.92 (0.88–0.96)*	0.94 (0.90–0.97)*	0.84 (0.81–0.87)*	0.88 (0.84–0.91)*	0.98 (0.94–1.02)	0.95 (0.91–0.99)*	0.96 (0.92–1.00)	0.91 (0.90–0.92)*
2022	5,297	4,216	4,531	4,671	4,885	5,101	5,791	5,379	4,809	5,134	5,147	5,978	60,939
IRR^c^ (95% CI)	0.91 (0.88–0.95)*	0.92 (0.88–0.96)*	0.97 (0.93–1.01)	0.99 (0.95–1.03)	0.98 (0.94–1.02)	1.07 (1.03–1.11)*	1.11 (1.07–1.15)*	0.94 (0.90–0.97)*	0.97 (0.93–1.01)	1.07 (1.03–1.11)*	1.06 (1.02–1.10)*	1.16 (1.12–1.21)*	1.01 (1.00–1.02)*
Changed hospital													
2019	514	428	436	394	498	471	478	529	472	506	526	502	5,754
2020	531	486	511	491	532	507	519	676	552	605	665	650	6,725
IRR^a^ (95% CI)	1.03 (0.91–1.17)	1.14 (1.00–1.30)*	1.17 (1.03–1.33)*	1.25 (1.09–1.43)*	1.07 (0.94–1.21)	1.08 (0.95–1.22)	1.09 (0.96–1.23)	1.28 (1.14–1.43)*	1.17 (1.03–1.33)*	1.20 (1.06–1.35)*	1.26 (1.13–1.42)*	1.29 (1.15–1.46)*	1.17 (1.13–1.21)*
2021	722	582	664	702	703	567	579	627	593	673	664	671	7,747
IRR^b^ (95% CI)	1.40 (1.25–1.58)*	1.36 (1.20–1.54)*	1.52 (1.35–1.72)*	1.78 (1.57–2.02)*	1.41 (1.26–1.59)*	1.20 (1.06–1.36)*	1.21 (1.07–1.37)*	1.19 (1.05–1.33)*	1.26 (1.11–1.42)*	1.33 (1.18–1.50)*	1.26 (1.12–1.42)*	1.34 (1.19–1.50)*	1.35 (1.30–1.39)*
2022	833	722	555	539	583	551	621	732	562	517	567	676	7,458
IRR^c^ (95% CI)	1.62 (1.45–1.81)*	1.69 (1.49–1.91)*	1.27 (1.12–1.45)*	1.37 (1.20–1.56)*	1.17 (1.04–1.32)*	1.17 (1.03–1.33)*	1.30 (1.15–1.47)*	1.38 (1.24–1.55)*	1.19 (1.05–1.35)*	1.02 (0.90–1.16)	1.08 (0.96–1.22)	1.35 (1.20–1.51)*	1.30 (1.25–1.34)*
Death													
2019	965	743	762	672	686	579	624	615	660	683	791	811	8,591
2020	900	821	792	659	672	603	608	622	621	704	767	891	8,660
IRR^a^ (95% CI)	0.93 (0.85–1.02)	1.10 (1.00–1.22)*	1.04 (0.94–1.15)	0.98 (0.88–1.09)	0.98 (0.88–1.09)	1.04 (0.93–1.17)	0.97 (0.87–1.09)	1.01 (0.90–1.13)	0.94 (0.84–1.05)	1.03 (0.93–1.15)	0.97 (0.88–1.07)	1.10 (1.00–1.21)*	1.01 (0.98–1.04)
2021	1,029	727	833	1,000	893	648	662	717	684	813	804	944	9,754
IRR^b^ (95% CI)	1.07 (0.98–1.17)	0.98 (0.88–1.09)	1.09 (0.99–1.21)	1.49 (1.35–1.64)*	1.30 (1.18–1.44)*	1.12 (1.00–1.25)*	1.06 (0.95–1.19)	1.17 (1.05–1.30)*	1.04 (0.93–1.16)	1.19 (1.07–1.32)*	1.02 (0.92–1.12)	1.16 (1.06–1.28)*	1.14 (1.10–1.17)*
2022	1,202	1,214	959	808	745	712	787	948	827	810	868	1,170	11,050
IRR^c^ (95% CI)	1.25 (1.14–1.36)*	1.63 (1.49–1.79)*	1.26 (1.14–1.39)*	1.20 (1.08–1.33)*	1.09 (0.98–1.21)	1.23 (1.10–1.37)*	1.26 (1.13–1.40)*	1.54 (1.39–1.71)*	1.25 (1.13–1.39)*	1.19 (1.07–1.31)*	1.10 (1.00–1.21)*	1.44 (1.32–1.58)*	1.29 (1.25–1.32)*

IRR, incident rate ratio; CI, confidence interval; a2020 versus 2019; b2021 versus 2019; c2022 versus 2019; *P <0.05.

The numbers of hospitalizations for COVID-19 (U) were 1,051 in 2020, 3,933 in 2021, and 12,791 in 2022. Significant increases were observed in 2021 and 2022, compared to 2020 (Table 5). The diagnostic classifications of the older emergency patients who were transported to hospitals for acute diseases and died thereafter are shown in Table 6. The numbers of deaths attributed to COVID-19 (U) were 102 in 2020, 568 in 2021, and 920 in 2022. Significant increases were observed in 2021 and 2022, compared to 2020. In terms of deaths caused by other diseases, the numbers of several diseases increased significantly in 2022 compared with 2019, including diseases of the circulatory system, diseases of the respiratory system, and diseases of the digestive system.

**Table 5:** Diagnoses of hospitalized patients transported to emergency departments in Osaka for acute diseases in 2019–2022.

Diagnoses (ICD-10), *n* (%)	2019	2020	IRR^a^ (95% CI)	2021	IRR^b^ (95% CI)	2022	IRR^c^ (95% CI)
Certain infectious and parasitic diseases (A + B)	3,578 (3.4)	3,663 (3.6)	1.02 (0.98–1.07)	3,826 (3.7)	1.07 (1.02–1.12)*	4,046 (3.5)	1.13 (1.08–1.18)*
Neoplasms (C)	6,380 (6.0)	6,203 (6.0)	0.97 (0.94–1.01)	6,442 (6.1)	1.01 (0.98–1.05)	6,309 (5.5)	0.99 (0.95–1.02)
Diseases of the blood and blood-forming organs and certain disorders involving the immune mechanism (D)	1,414 (1.3)	1,455 (1.4)	1.03 (0.96–1.11)	1,463 (1.4)	1.03 (0.96–1.11)	1,444 (1.3)	1.02 (0.95–1.10)
Endocrine, nutritional, and metabolic diseases (E)	5,218 (4.9)	5,142 (5.0)	0.99 (0.95–1.02)	4,784 (4.6)	0.92 (0.88–0.95)*	5,104 (4.4)	0.98 (0.94–1.02)
Mental and behavioral disorders (F)	478 (0.5)	426 (0.4)	0.89 (0.78–1.02)	317 (0.3)	0.66 (0.57–0.77)*	306 (0.3)	0.64 (0.55–0.74)*
Diseases of the nervous system (G)	3,924 (3.7)	3,563 (3.5)	0.91 (0.87–0.95)*	3,373 (3.2)	0.86 (0.82–0.90)*	3,310 (2.9)	0.84 (0.81–0.88)*
Diseases of the eye and adnexa (H00-59)	24 (0.0)	33 (0.0)	1.38 (0.79–2.43)	16 (0.0)	0.67 (0.33–1.31)	23 (0.0)	0.96 (0.52–1.77)
Diseases of the ear and mostoid process (H60-96)	2,088 (2.0)	1,950 (1.9)	0.93 (0.88–0.99)*	1,794 (1.7)	0.86 (0.81–0.92)*	1,682 (1.5)	0.81 (0.76–0.86)*
Diseases of the circulatory system (I)	25,753 (24.1)	25,371 (24.7)	0.99 (0.97–1.00)	25,404 (24.2)	0.99 (0.97–1.00)	26,112 (22.7)	1.01 (1.00–1.03)*
Diseases of the respiratory system (J)	22,694 (21.2)	19,227 (18.7)	0.85 (0.83–0.86)*	18,619 (17.8)	0.82 (0.80–0.84)*	19,238 (16.7)	0.85 (0.83–0.86)*
Diseases of the digestive system (K)	13,135 (12.3)	12,812 (12.5)	0.98 (0.95–1.00)	13,114 (12.5)	1.00 (0.97–1.02)	13,232 (11.5)	1.01 (0.98–1.03)
Diseases of the skin and subcutaneous tissue (L)	1,012 (1.0)	1,062 (1.0)	1.05 (0.96–1.14)	1,070 (1.0)	1.06 (0.97–1.15)	1,084 (0.9)	1.07 (0.98–1.17)
Diseases of the musculoskeletal system and connective tissue (M)	3,213 (3.0)	3,246 (3.2)	1.01 (0.96–1.06)	3,020 (2.9)	0.94 (0.89–0.99)*	2,909 (2.5)	0.91 (0.86–0.95)*
Diseases of the genitourinary system (N)	7,647 (7.2)	8,297 (8.1)	1.09 (1.05–1.12)*	8,742 (8.3)	1.14 (1.11–1.18)*	8,549 (7.4)	1.12 (1.08–1.15)*
Pregnancy, childbirth, the puerperium, certain conditions originating in the perinatal period, congenital malformations, deformations, and chromosomal abnormalities (O+P+Q)	31 (0.0)	39 (0.0)	1.26 (0.76–2.09)	51 (0.1)	1.65 (1.03–2.66)*	48 (0.0)	1.55 (0.97–2.52)
Symptoms, signs and abnormal clinical and laboratory findings, not elsewhere classified (R)	4,439 (4.2)	3,644 (3.6)	0.82 (0.79–0.86)*	3,333 (3.2)	0.75 (0.72–0.79)*	3,200 (2.8)	0.72 (0.69–0.75)*
Injury, poisoning, and certain other consequences of external causes (S + T)	5,329 (5.0)	5,454 (5.3)	1.02 (0.99–1.06)	5,442 (5.2)	1.02 (0.98–1.06)	5,707 (5.0)	1.07 (1.03–1.11)*
Others (U)	0 (0.0)	1,051 (1.0)	NA	3,933 (3.8)	3.74 (3.50–4.01)*	12,791 (11.1)	12.17 (11.43–12.97)*
External causes of morbidity and mortality (V + W + X + Y)	82 (0.1)	41 (0.0)	0.50 (0.33–0.74)*	56 (0.1)	0.68 (0.48–0.97)*	25 (0.0)	0.30 (0.19–0.48)*
Factors influencing health status and contact with health services (Z)	37 (0.0)	23 (0.0)	0.62 (0.35–1.07)	19 (0.0)	0.51 (0.28–0.92)*	24 (0.0)	0.65 (0.37–1.11)
Unknown	355 (0.3)	32 (0.0)	0.09 (0.06–0.13)*	31 (0.0)	0.09 (0.06–0.13)*	31 (0.0)	0.09 (0.06–0.13)*

ICD-10, international statistical classification of diseases and related health problems, 10th revision; IRR, incident rate ratio; CI, confidence interval; NA, not assessed. Others (U) represents COVID-19. ^a^2020 versus 2019. ^b^2021 versus 2019 except for Others (U). ^b^2021 versus 2020 for Others (U). ^c^2022 versus 2019 except for Others (U). ^c^2022 versus 2020 for Others (U). *P < 0.05.

**Table 6:** Diagnoses of patients transported to emergency departments in Osaka for acute diseases and died following hospitalization in 2019–2022.

Diagnoses (ICD-10), *n* (%)	2019	2020	IRR^a^ (95% CI)	2021	IRR^b^ (95% CI)	2022	IRR^c^ (95% CI)
Certain infectious and parasitic diseases (A + B)	396 (4.6)	434 (5.0)	1.10 (0.95–1.26)	564 (5.8)	1.42 (1.25–1.62)*	582 (5.3)	1.47 (1.29–1.67)*
Neoplasms (C)	1,742 (20.3)	1,697 (19.6)	0.97 (0.91–1.04)	1,799 (18.4)	1.03 (0.97–1.10)	1,716 (15.5)	0.99 (0.92–1.05)
Diseases of the blood and blood-forming organs and certain disorders involving the immune mechanism (D)	170 (2.0)	181 (2.1)	1.06 (0.86–1.32)	185 (1.9)	1.09 (0.88–1.35)	183 (1.7)	1.08 (0.87–1.33)
Endocrine, nutritional, and metabolic diseases (E)	171 (2.0)	199 (2.3)	1.16 (0.94–1.44)	197 (2.0)	1.15 (0.93–1.42)	249 (2.3)	1.46 (1.19–1.78)*
Mental and behavioral disorders (F)	7 (0.1)	3 (0.0)	0.43 (0.07–1.88)	0 (0.0)	NA	2 (0.0)	0.29 (0.03–1.50)
Diseases of the nervous system (G)	81 (0.9)	97 (1.1)	1.20 (0.88–1.63)	101 (1.0)	1.25 (0.92–1.69)	110 (1.0)	1.36 (1.01–1.83)*
Diseases of the eye and adnexa (H00-59)	0 (0.0)	0 (0.0)	NA	1 (0.0)	NA	1 (0.0)	NA
Diseases of the ear and mostoid process (H60-96)	0 (0.0)	1 (0.0)	NA	1 (0.0)	NA	0 (0.0)	NA
Diseases of the circulatory system (I)	2,875 (33.5)	2,762 (31.9)	0.96 (0.91–1.01)	2,913 (29.9)	1.01 (0.96–1.07)	3,173 (28.7)	1.10 (1.05–1.16)*
Diseases of the respiratory system (J)	2,000 (23.3)	2,056 (23.7)	1.03 (0.97–1.09)	2,108 (21.6)	1.05 (0.99–1.12)	2,598 (23.5)	1.30 (1.22–1.38)*
Diseases of the digestive system (K)	468 (5.5)	474 (5.5)	1.01 (0.89–1.15)	571 (5.9)	1.22 (1.08–1.38)*	646 (5.9)	1.38 (1.22–1.56)*
Diseases of the skin and subcutaneous tissue (L)	22 (0.3)	8 (0.1)	0.36 (0.14–0.85)*	17 (0.2)	0.77 (0.39–1.52)	31 (0.3)	1.41 (0.79–2.55)
Diseases of the musculoskeletal system and connective tissue (M)	32 (0.4)	25 (0.3)	0.78 (0.44–1.36)	40 (0.4)	1.25 (0.77–2.06)	44 (0.4)	1.38 (0.85–2.24)
Diseases of the genitourinary system (N)	244 (2.8)	287 (3.3)	1.18 (0.99–1.40)	341 (3.5)	1.40 (1.18–1.65)*	386 (3.5)	1.58 (1.34–1.86)*
Pregnancy, childbirth, the puerperium, certain conditions originating in the perinatal period, congenital malformations, deformations, and chromosomal abnormalities (O+P+Q)	1 (0.0)	1 (0.0)	1.00 (0.01–78.50)	0 (0.0)	NA	4 (0.0)	4.00 (0.40–196.99)
Symptoms, signs and abnormal clinical and laboratory findings, not elsewhere classified (R)	231 (2.7)	185 (2.1)	0.80 (0.66–0.98)*	206 (2.1)	0.89 (0.74–1.08)	217 (2.0)	0.94 (0.78–1.14)
Injury, poisoning, and certain other consequences of external causes (S + T)	128 (1.5)	143 (1.7)	1.12 (0.87–1.43)	137 (1.4)	1.07 (0.83–1.37)	182 (1.7)	1.42 (1.13–1.80)*
Others (U)	0 (0.0)	102 (1.2)	NA	568 (5.8)	5.57 (4.50–6.94)*	920 (8.3)	9.02 (7.34–11.18)*
External causes of morbidity and mortality (V + W + X + Y)	2 (0.0)	0 (0.0)	NA	2 (0.0)	1.00 (0.07–13.80)	1 (0.0)	0.50 (0.01–9.60)
Factors influencing health status and contact with health services (Z)	1 (0.0)	2 (0.0)	2.00 (0.10–117.99)	1 (0.0)	1.00 (0.01–78.50)	2 (0.0)	2.00 (0.10–117.99)
Unknown	20 (0.2)	3 (0.0)	0.15 (0.03–0.51)*	2 (0.0)	0.10 (0.01–0.41)*	3 (0.0)	0.15 (0.03–0.51)*

ICD-10, international statistical classification of diseases and related health problems, 10th revision; IRR, incident rate ratio; CI, confidence interval; NA, not assessed. Others (U) represents COVID-19. ^a^2020 versus 2019. ^b^2021 versus 2019 except for Others (U). ^b^2021 versus 2020 for Others (U). ^c^2022 versus 2019 except for Others (U). ^c^2022 versus 2020 for Others (U). *P < 0.05.

## 4 Discussion

Using the population-based ORION registry, this descriptive study revealed the reality regarding older patients transported by ambulance to emergency departments in Osaka Prefecture, Japan in 2019–2022. The number of emergency patients, number of emergency patients transported for acute diseases, and the number of emergency patients transported for acute diseases who were later hospitalized all decreased in 2020 and 2021 compared with 2019, but were higher in 2022. This population-based descriptive study regarding the influence of the COVID-19 pandemic on EMS in older patients is expected to prove useful for healthcare systems and policy planning.

The number of emergency transports increased in 2022 vs. 2019, with the majority being for acute diseases. The number of older emergency patients transported because of acute diseases decreased in 2020 and 2021, but increased in 2022. A similar trend was observed during the Severe Acute Respiratory Syndrome outbreak in 2003 (20). This change might have been related to a return to normal social life, including the removal of behavioral restrictions, fewer people wearing masks, and an increase in the number of patients with COVID-19.

However, the number of difficulties obtaining acceptance from hospitals for emergency patients transported for acute disease gradually increased after the COVID-19 pandemic. The number of older emergency patients who were transported for acute diseases and later died in hospital also increased in 2021 and 2022 vs. 2019. In particular, the number of deaths from cardiovascular, respiratory, and gastrointestinal diseases increased in 2022. Even before the COVID-19 pandemic, emergency department crowding was reported to be related to increased deaths (21). A previous Japanese study showed that this increase in older emergency patients had a direct significant negative effect on emergency department overcrowding (22). Several studies have reported that patient outcomes were affected by the COVID-19 pandemic (7, 23–25). The pandemic reportedly increased the number of cases that were difficult to transport, the time required for initial responses from EMSs, and caused some patients to refrain from seeking medical care over fear of COVID-19 infection (7, 26–28). Older patients are more vulnerable to health problems; therefore, these factors may have increased the number of people who became seriously ill and worsened their prognoses. The time intervals from ambulance call to arrival on scene increased over year in this study. However, the definitive reasons for the increase in the number of deaths following emergency hospitalization after the COVID-19 pandemic are unclear, because data regarding emergency procedures, particularly for cardiovascular, respiratory, and gastrointestinal diseases after hospital arrival, were unavailable for this study. It is essential to continue monitoring changes in the number of emergency patients transported to hospitals, as well as their number of deaths. Regular assessments of the impact of the COVID-19 pandemic on the EMS system can be used to formulate EMS system plans for future pandemics and disasters. Based on the lessons learned from this experience, it would be useful to prepare for future pandemics by, for example, expanding consultation services that can be used before calling for EMS, educating residents on the proper use of ambulances, and establishing a task force within the government to respond to such situations.

In our study, the number of hospitalizations and deaths caused by COVID-19 among older emergency patients significantly increased over the study period. In 2022, when the Omicron variant of the SARS-CoV-2 virus became dominant, the mortality rate of COVID-19 declined among all patients, including older ones (29). On the other hand, the number of confirmed COVID-19 cases increased significantly in Osaka Prefecture, from 30,000 in 2020 to 170,000 in 2021, then 2,340,000 in 2022 (30). The increase in the number of hospitalizations and deaths caused by COVID-19 in this study was likely attributable to the greater impact of the increase in the number of COVID-19 cases. Older patients with COVID-19 accounted for 10% of all hospitalized patients in 2022, placing a significant overall burden on EMS. Further studies are warranted to determine the long-term transition characteristics of older patients with COVID-19 who are transported to emergency hospitals.

This study was subject to several key limitations worth noting. First, the ORION registry records patient data concerning patients who are transported to emergency hospitals and emergency critical care centers in Osaka Prefecture but does not register data regarding emergency patients who are transported to non-emergency medical institutions in the prefecture or to medical institutions outside of the prefecture. Second, we had no information on disease severity or the treatments received by our cohort of emergency patients within the hospitals they were admitted to. Finally, patient-related factors such as medical history, and social factors such as the person who called for EMS, hospital capacity, and regional differences in EMS operations, may influence patient prognoses; however, these factors were not included in this study (31–33).

## 5 Conclusion

The number of older patients transported to emergency departments in Osaka Prefecture for acute diseases decreased in 2020 and 2021, but increased in 2022. The number of difficulties obtaining patient acceptance from hospitals for these patients increased as COVID-19 spread through the region. By 2022, the number of deaths from acute diseases, including cardiovascular, respiratory, and gastrointestinal diseases, also increased. Continuous monitoring will be necessary to assess future changes.

## Data Availability

The data analyzed in this study is subject to the following licenses/restrictions: the data supporting the findings of this study are available from the Osaka Prefectural government; however, the availability of these data is restricted. The data cannot be shared publicly because of the Protection Ordinance for Personal Information law in Osaka Prefecture. They may be accessible to qualified researchers who apply for access and are approved by the technical committee. Requests to access these datasets should be directed to https://www.pref.osaka.lg.jp/o100030/iryo/qq/orion_teikyo.html.
